# Rapid nitrification involving comammox and canonical *Nitrospira* at extreme pH in saline‐alkaline lakes

**DOI:** 10.1111/1462-2920.16337

**Published:** 2023-02-03

**Authors:** Anne Daebeler, Queralt Güell‐Bujons, Maria Mooshammer, Thomas Zechmeister, Craig W. Herbold, Andreas Richter, Michael Wagner, Holger Daims

**Affiliations:** ^1^ University of Vienna Centre for Microbiology and Environmental Systems Science, Division of Microbial Ecology Vienna Austria; ^2^ Biology Centre CAS, Budweis Institute of Soil Biology and Biogeochemistry Czechia; ^3^ Institut de Ciències del Mar (ICM‐CSIC), Passeig Marítim de la Barceloneta 37‐49 Barcelona Catalonia Spain; ^4^ Centre for Microbiology and Environmental Systems Science, Division of Terrestrial Ecosystem Research University of Vienna Vienna Austria; ^5^ Biological Station Lake Neusiedl Illmitz Austria; ^6^ The Comammox Research Platform University of Vienna Vienna Austria; ^7^ Center for Microbial Communities, Department of Chemistry and Bioscience Aalborg University Aalborg Denmark

## Abstract

Nitrite‐oxidizing bacteria (NOB) catalyse the second nitrification step and are the main biological source of nitrate. The most diverse and widespread NOB genus is *Nitrospira*, which also contains complete ammonia oxidizers (comammox) that oxidize ammonia to nitrate. To date, little is known about the occurrence and biology of comammox and canonical nitrite oxidizing *Nitrospira* in extremely alkaline environments. Here, we studied the seasonal distribution and diversity, and the effect of short‐term pH changes on comammox and canonical *Nitrospira* in sediments of two saline, highly alkaline lakes. We identified diverse canonical and comammox *Nitrospira* clade A‐like phylotypes as the only detectable NOB during more than a year, suggesting their major importance for nitrification in these habitats. Gross nitrification rates measured in microcosm incubations were highest at pH 10 and considerably faster than reported for other natural, aquatic environments. Nitrification could be attributed to canonical and comammox *Nitrospira* and to *Nitrososphaerales* ammonia‐oxidizing archaea. Furthermore, our data suggested that comammox *Nitrospira* contributed to ammonia oxidation at an extremely alkaline pH of 11. These results identify saline, highly alkaline lake sediments as environments of uniquely strong nitrification with novel comammox *Nitrospira* as key microbial players.

## INTRODUCTION

Nitrite oxidation, the second step of nitrification, is a key process of the global nitrogen cycle occurring in nearly all oxic habitats including soil, freshwater and marine ecosystems, engineered environments, and geothermal springs (Daims et al., 2016). In most terrestrial environments, uncultured members of the genus *Nitrospira* are the dominant known NOB (Daims et al., 2016). Over the past 7 years, *Nitrospira* representatives have been recognized as metabolically flexible organisms able to support growth via the oxidation of hydrogen or formate next to nitrite (Koch et al., 2014, 2015) and were most recently also identified as complete ammonia oxidizers (comammox) (Daims et al., 2015; van Kessel et al., 2015). Owing to their late discovery, the environmental distribution and contribution to nitrification of comammox *Nitrospira* is only beginning to be understood (Li et al., 2019; Pjevac et al., 2017; Shi et al., 2020; Wang et al., 2019; Xia et al., 2018).

In previous studies, we obtained evidence, by molecular and cultivation‐based approaches, for the presence of canonical nitrite‐oxidizing *Nitrospira* and of comammox *Nitrospira* in sediments of Austrian hypertrophic, saline‐alkaline lakes (Daebeler et al., 2020; Pjevac et al., 2017). Diverse *Nitrospira* communities were found in samples from nine lakes in the national park ‘Neusiedler See‐Seewinkel’, and enriched canonical nitrite‐oxidizing *Nitrospira* strains catalysed nitrite oxidation at exceptionally high pH values up to 10.5 (Daebeler et al., 2020). However, the functional importance of comammox *Nitrospira* in these lake systems remained unexplored. The evolution of the saline‐alkaline lakes is related to the lack of alkaline earth cations compared with the richness of bicarbonate, a shallow depressed structure with a closed non‐leaking drainage basin, and an evaporation rate that exceeds the inflow (Boros et al., 2014). Comparatively low levels of cations result in the water bodies being a weakly buffered solution with strong daily and seasonal pH fluctuations (Boros et al., 2017). A combination of salinity and alkalinity in these systems poses multiple stressors to prokaryotes (and other life), such as damage to proteins, reversal of the transmembrane pH gradient needed for building up proton motive force, reduced availability of micro‐ and macro‐nutrients, and osmotic stress (Banciu & Sorokin, 2013). The activity range of the cultured *Nitrospira* strains, including comammox *Nitrospira*, collectively reaches from a pH of 3 to 10.2, with the single isolates and enrichments displaying more narrow ranges (Blackburne et al., 2007; Daebeler et al., 2020; Ehrich et al., 1995; Takahashi et al., 2020; Wegen et al., 2019). The highest known tolerated salinity for cultured canonical *Nitrospira* lies at sea water level with about 35 g/L (Haaijer et al., 2013; Watson et al., 1986). Strikingly, comammox *Nitrospira* have not been detected in such highly saline systems yet.

Here, we assessed the seasonal community dynamics, and the response to short‐term pH changes, of canonical and comammox *Nitrospira* in sediments from two hypertrophic saline‐alkaline lakes in the national park ‘Neusiedler See‐Seewinkel’: lake Unterer Stinkersee (US) and lake Herrensee (HS), which differ in a range of chemical and ecological properties. Lake US is classified as an alkaline type saline‐alkaline lake with Na^+^ and HCO_3_^−^ as the only abundant ions, whereas lake HS classifies as a sulphate type with HCO_3_^−^ as the most and SO_4_^−^ as the second most abundant anion present (Boros et al., 2014). The water of lake HS usually lies within the hyposaline range (max. ~7 g salts/L) with a moderately alkaline pH (max. pH 8.5), whereas lake US is subsaline (max. ~4 g salts/L) but shows high alkalinity (max. pH 9.7) (Boros et al., 2014; Krachler et al., 2012). Furthermore, the two lakes each represent one of the two ecological types of saline‐alkaline lakes found in the national park. Lake US is of a ‘turbid–white’ type, which is dominated by large amounts of suspended inorganic (clay) particles that generally cause high turbidity and a light grey‐white colour of the water. Lake HS is of a ‘non‐turbid–coloured’ type, which has much less suspended particles and is rather dominated by dissolved brown‐black coloured (humic) substance (Krachler et al., 2012). We hypothesized that (i) fluctuations in pH influence the NOB and comammox community structures; (ii) highest gross nitrification rates occur at the pH level closest to the average in situ pH of the lakes; (iii) different subsets of the nitrifier populations are active at different pH levels, indicating niche partitioning.

The community composition of sediment canonical and comammox *Nitrospira* was monitored for 15 months and related to seasonal dynamics of physico‐chemical factors measured in the lakes. Additionally, a short‐term microcosm experiment with lake sediment samples was performed with different pH regimes ranging from neutral to extremely alkaline. The gross nitrate production rates were determined at all pH levels, and the nitrifier communities and activities were analysed by amplicon sequencing of protein‐coding nitrifier marker genes and their transcripts.

## MATERIALS AND METHODS

### Monthly sampling of lake sediments and microcosm incubations

For a seasonal monitoring (from April 2014 to June 2015) of canonical and comammox *Nitrospira* communities, four replicate sediment cores per lake were collected, once per month, from lake Herrensee (HS) and lake Unterer Stinkersee (US) of the national park ‘Neusiedler See‐Seewinkel’. The replicate cores were taken from the same spot each month, about 20 m into the lake and within a 10 × 10 m area. A map of the sampling area depicting the two lakes is shown in Figure S1. At all sampling times, water temperature, pH, and conductivity were measured with a SenTix 41 electrode and a Multiline field instrument with a TetraCon 325 (WTW). Salinity was inferred from conductivity using a previously established, experimentally verified conversion factor (Boros et al., 2014). After immediate, cooled transport to the lab, the upper sediment layer (depth: 0–10 cm) was separated from the core with a sterile spatula and homogenized. Upper sediment layer material from one of the four replicate cores was used for pore water measurements of ammonium‐N, nitrite, and nitrate‐N. The material from the remaining three cores was stored at −20°C for molecular analysis.

Three replicate sediment cores and approximately 1 L of surface water for microcosm incubations were collected in November 2014 from each lake. Lake water for the incubations and to be used as matrix for isotope ratio mass spectrometry (IRMS) measurements was centrifuged for 20 min at 18,000 × *g* and sterilized by filtration (pore size 0.2 μm). Approximately 50 g of sediment from the top 10 cm of each core was centrifuged at 18,000 × *g* for 10 min to remove excess water before sieving the sediment through a 1 mm sieve, mixing, and homogenizing. Ten grams of sediment was then transferred to a sterile 250 mL flask, supplemented with 30 mL sterile filtered lake water, and shaken at 150 rpm for 1 h to homogenize the slurry. For each lake and each pH regime (pH 7.6, 9.0, 10.0, and 11.0), four replicate bottles were then adjusted to the respective pH by titration with either 2 M HCl or 2 M NaOH. The lake sediment slurries were incubated in the dark at 28°C with agitation (150 rpm) for 7 days. All treatments were monitored and pH values adjusted, if necessary, twice every 24 h during the experiment (Figure S2). The bottles were closed with screw caps during the preparatory steps and the incubation. For the determination of gross nitrification rates, we added 500 μL of ^15^ N—NO_3_^−^ (33 at%) on day six of the incubation. The amount of added ^15^ N—NO_3_^−^ was 1%–3% of the nitrate concentration in the incubations on that day. For the analyses of nucleic acids, samples were taken from the homogenized sediment before microcosm set‐up and from the sediment slurries at the end of the incubation.

### Determination of inorganic N compounds, isotopic signatures and quantification of gross nitrification rates

Measurements of ammonium, nitrite and nitrate with the monthly samples were conducted on extracted sediment pore water as follows. Approximately 500 g of sediment from the top 10 cm of each core was centrifuged at 4000 × *g* for 20 min at 4°C to receive the interstitial water for analysis of ammonium‐N, nitrite and nitrate‐N within 12 h. The determination of ammonium‐nitrogen was performed after the German standard methods for the examination of water, waste water and sludge; cations (group E) (DIN 38406‐5:1983‐10). The concentration of nitrite was measured using the molecular absorption spectrometric method described in ISO 6777:1984 (OENORM EN 26777:1993). The analysis of nitrate was determined spectrometrically with sulfosalicylic acid after the German standard methods for the examination of water, waste water and sludge—anions (group D)—Part 29 (DIN 38405‐29:1994‐11).

Samples for the photometric determination of inorganic N compound concentrations were taken daily from each microcosm. We used the Griess assay according to Miranda et al. (2001) for measuring nitrite and nitrate, and the phenate reaction according to Kandeler and Gerber (1988) for measuring ammonium.

For the quantification of gross nitrification rates, samples were taken 2.5, 24, and 48 h after ^15^ N—NO_3_^−^ addition on Day 6. Isotopic composition of NO_3_^−^ was determined by a method based on the reduction of NO_3_^−^ to N_2_O via NO_2_^−^ by using azide under acidified conditions following the protocol of Lachouani et al. (2010). Briefly, 250 μL sample or standard was transferred to 12‐mL exetainer. After purging the vials with helium to eliminate air‐N_2_O in the sample headspace, 250 μL of 50 mM VCl_3_ (in 1 M HCl solution) and 50 μL 1 M sodium azide (in 10% acetic acid solution) were injected and the vials were placed on a shaker at 37 °C for 24 h. The reaction was stopped by injecting 100 μL of 6 M NaOH. For mass calibration, NO_3_^−^ standards ranging from natural abundance to 10 at% were analysed. N_2_O concentration and isotopic ratio of the azide conversion were determined using a purge‐and‐trap GC/IRMS system (PreCon, GasBench II headspace analyser, Delta Advantage V; Thermo Fischer, Vienna, Austria). Gross rates of nitrification were calculated based on isotope pool dilution theory (Kirkham & Bartholomew, 1954) as established by Wanek et al. (2010) using the changes in nitrate concentration and in the δ^15^N—NO_3_ values between pairs of time points. The values used for the calculation are given in Table S1.

### DNA and RNA extraction, cDNA synthesis, amplicon library preparation, NGS sequencing, and sequence analysis

DNA from the triplicate seasonal samples was extracted with the Fast DNA Spin Kit for Soil (MP BIO) following the manufacturer's protocol. The seasonal DNA samples were screened for the presence of *Nitrospira*, *Nitrobacter*, *Nitrococcus*, *Nitrospinaceae*, *Nitrotoga*, comammox *Nitrospira* clades A and B, beta‐ and gammaproteobacterial ammonia‐oxidizing bacteria (AOB), and ammonia‐oxidizing archaea (AOA) by PCR. Primers, target genes, and PCR conditions are summarized in Table S2.

Total nucleic acids from the quadruplicate samples of the microcosm incubations were extracted according to Angel and Conrad (2013), purified using the One stop PCR inhibitor removal kit (Zymo Research), and stored at −20°C. Sub‐samples of 10–42 μL were taken no longer than 2 months post nucleic acid extraction and treated with DNA‐free TURBO DNase (Life) according to the manufacturer's instructions in the presence of an RNase inhibitor (RNase OUT, Invitrogen). RNA was then purified using the GeneJET RNA Cleanup and Concentration Micro Kit (Thermo Scientific) according to manufacturers' instructions and eluted in RNA storage solution (Life). All samples were checked for DNA contamination by attempting to PCR amplify the nitrite oxidoreductase subunit beta (*nxrB*) gene of *Nitrospira*, the most abundant NOB group according to the DNA analyses, and subsequent gel electrophoresis as described below. Only DNA‐negative samples were used for cDNA synthesis, whereas others were repeatedly subjected to DNase treatments until DNA was not detectable. Reverse transcription was performed using random hexamer primers (Thermo Scientific), an RNase inhibitor (RNase OUT, Invitrogen), and the Superscript IV reverse transcriptase Kit (Invitrogen) according to the manufacturer's instructions. Cycling conditions for reverse transcription were as follows: 25°C for 5 min followed by 42°C for 60 min and 70°C for 15 min.

All DNA and cDNA samples were stored at −20°C. For the gene‐based analysis of NOB in the seasonal samples, we targeted *Nitrospira nxrB* and *Nitrospira* ammonia monooxygenase subunit A (*amoA*). For the gene‐ and transcript‐based analyses of the microcosm experiment, we additionally targeted *Nitrososphaerales amoA*. Amplicon library generation and Illumina MiSeq sequencing was performed as described by Herbold et al. (2015) with the exception of using the QIAquick PCR purification Kit (Qiagen) for purification of amplicons of the expected length. Alternatively, the QIAquick Gel extraction Kit (Qiagen) was used to purify PCR products of the correct length from agarose gels if additional, unspecific amplification products of different lengths were present.

Paired end reads were processed and mapped to operational taxonomic unit (OTU) representatives as described by Herbold et al. (2015) including chimera checks using the UPARSE pipeline (Edgar, 2010, 2013). Bioinformatic processing of *nxrB* read data was performed as described by Daebeler et al. (2020). The sequence identity thresholds applied for OTU clustering of *amoA* gene sequences were 87% for *Nitrososphaerales amoA* (Pester et al., 2011) and 95% for comammox *amoA* (Pjevac et al., 2017). Non‐target sequences were identified by alignment to reference sequences using the blastx tool of the NCBI website (National Center for Biotechnology Information (NCBI), 1988) and excluded from further analysis. Amplicon sequence data of all samples were normalized using the GMPR function (Chen et al., 2018) with a minimal intersect of two species per sample.

The raw, demultiplexed fastq files were deposited at the European Nucleotide Archive (ENA) database under study accession numbers PRJEB47424, PRJEB47446, PRJEB47390, PRJEB47406, and PRJEB47407 for the data sets of *amoA* of comammox *Nitrospira* from seasonal samples, *nxrB* of canonical and comammox *Nitrospira* from seasonal samples, *amoA* of comammox *Nitrospira* from the pH‐controlled incubations, *nxrB* of canonical and comammox *Nitrospira* from the pH‐controlled incubations, and *amoA* of *Nitrososphaerales* from the pH‐controlled incubations, respectively.

### Phylogenetic analyses

*Nitrospira nxrB* nucleotide sequences and *Nitrospira* and *Nitrososphaerales* AmoA protein sequences (obtained by in silico translation of *amoA* gene sequences) from this study were aligned with selected reference sequences using mafft‐linsi v.7.312 (Katoh et al., 2002) and trimmed using Trimal v1.4.rev15 (Capella‐Gutiérrez et al., 2009) with option ‐automated1. The resulting alignments consisting of 474, 301, and 386 columns for *Nitrospira nxrB*, *Nitrospira* AmoA, and *Nitrososphaerales* AmoA, respectively, were used to calculate trees in IQ‐TREE v1.6.2 (Nguyen et al., 2015) with default settings including 1000 bootstrap iterations. IQ‐TREE calculations further included model prediction by ModelFinder (Kalyaanamoorthy et al., 2017), which identified the best‐fit models to be GTR + F + I + G4, LG + F + G4, and mtZOA + G4 for *Nitrospira nxrB*, *Nitrospira* AmoA, and *Nitrososphaerales* AmoA, respectively.

### Statistical analyses

Statistical analyses were performed using R version 3.4.4 (R Core Team, 2013). Environmental factors measured in this study were centred and/or scaled using the function ‘scale’. Permutational multivariate analyses were performed to assess significant differences in the *Nitrospira nxrB* and *amoA* gene‐based communities between monthly samples with the function ‘adonis’. To factor out time as an underlying influence on community differences, it was treated as a random factor by including it in the first position of the model formula (i.e. nxrB_community ~ time + pH * salinity). A constrained principal coordinates analysis was performed to assess significant differences in the *Nitrospira nxrB* and *amoA* gene‐ and transcript‐based communities between samples from the microcosm incubations with the function ‘capscale’. Both analyses were based on Bray–Curtis distance matrices and the functions are a part of the package vegan (Oksanen et al., 2010). Autocorrelation of the environmental data was checked with the Durbin–Watson test using the ‘durbinWatsonTest’ function from the car package (Fox & Weisberg, 2019). Differences in gross nitrification rates were checked for significance by Tukey's honesty significant difference (HSD) test.

## RESULTS

### Seasonal dynamics of environmental conditions and canonical/comammox *Nitrospira* community structures

**FIGURE 1:**
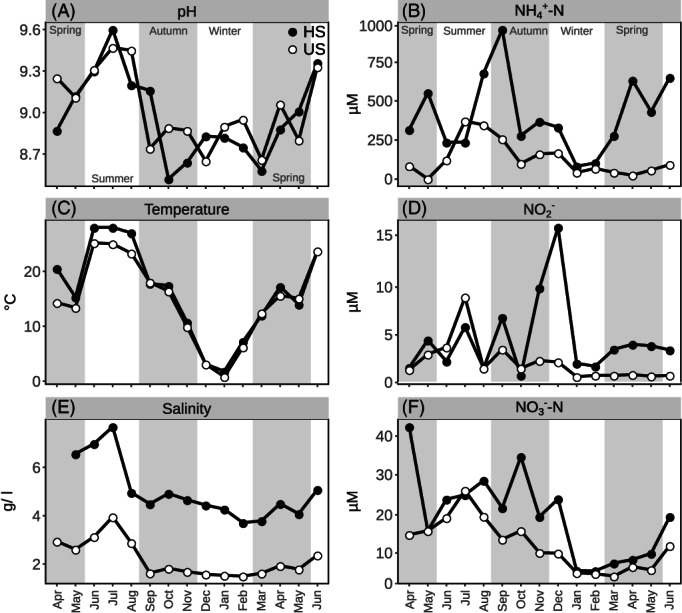
Environmental parameters measured for lake water (A, C, E) and sediment pore water (B, D, F) in monthly samples taken from April 2014 to June 2015 from the two studied saline‐alkaline lakes HS (lake Herrensee) and US (lake Unterer Stinkersee).

To obtain insights into the seasonal dynamics of nitrite oxidizer and comammox populations in a saline‐alkaline system, we first monitored selected environmental conditions in the water and top sediment of two saline‐alkaline lakes during a 15 months period from April 2014 to June 2015. During the observation period, temperature, pH, and salinity dropped considerably in autumn and winter compared to spring and summer (Figure 1A,C,E). Temperature and pH were generally comparable between the two lakes, but salinity was higher in lake HS throughout the entire observation period. The concentrations of inorganic N species occasionally rose to higher levels in lake HS (Figure 1B,D,F).

We then screened replicate samples from sediment of both lakes at each season for the stable presence of canonical and comammox *Nitrospira*, as well as for members of the NOB lineages *Nitrobacter*, *Nitrococcus*, *Nitrospinaceae*, and *Nitrotoga* by PCR targeting *nxrB* and comammox *amoA* genes. Only canonical and comammox *Nitrospira* were detectable by this approach, and the analysis was continued by *Nitrospira nxrB* and *amoA* gene amplicon sequencing.

**FIGURE 2:**
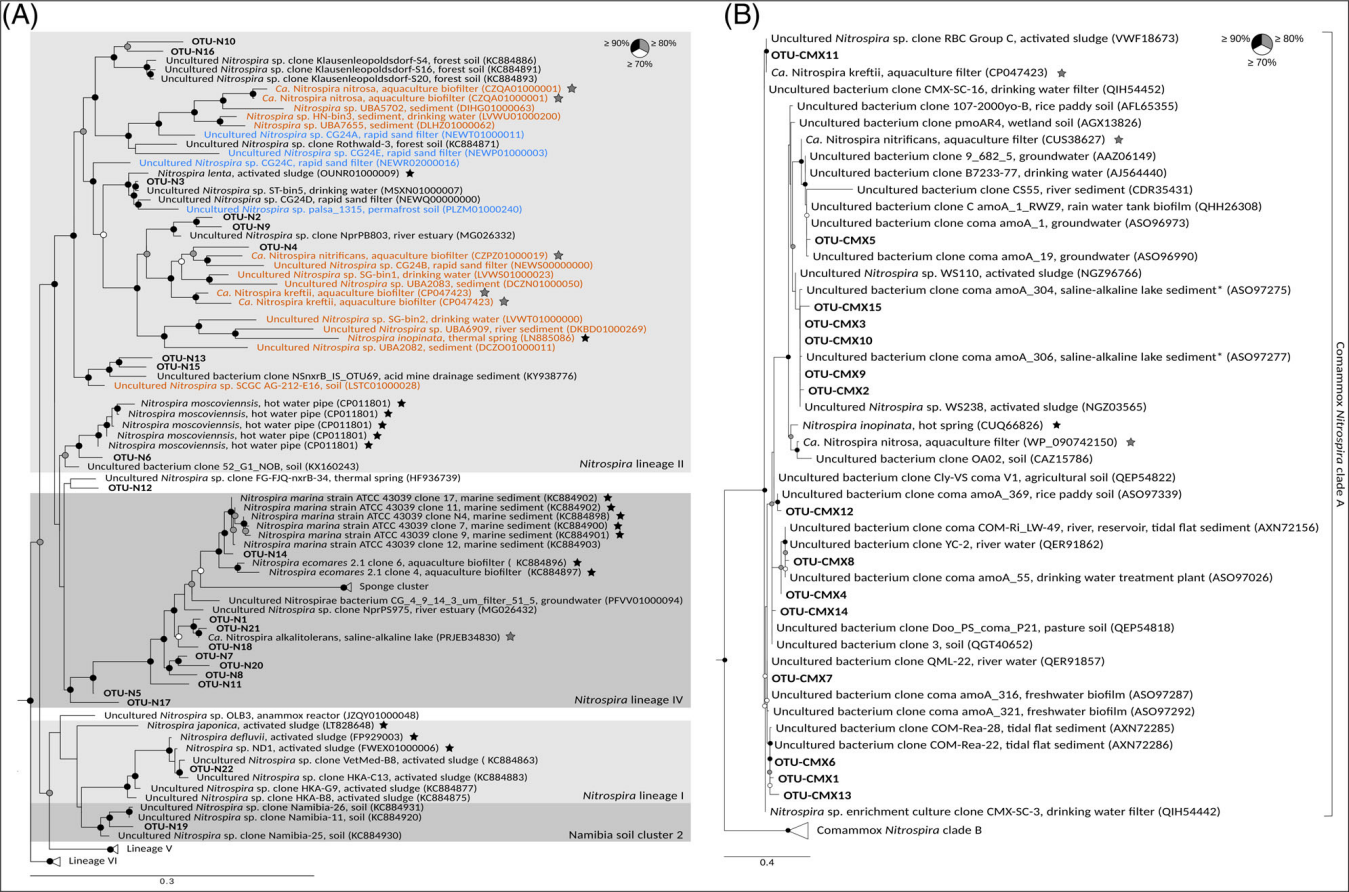
Phylogenetic maximum likelihood analysis of canonical and comammox Nitrospira in lakes HS and US. The trees show the affiliations of *Nitrospira nxrB* (A) sequences and *Nitrospira* AmoA (B) sequences, which were retrieved from monthly samples and from sediment microcosms, to reference sequences. Sequences obtained from in this study are printed in bold. The *nxrB* gene sequences of cultured cultured *Brocadia, Jettenia, Kuenenia, Scalindula*, and *Nitrospina* species and the AmoA gene sequences of cultured *Nitrosomonas, Nitrososphaera*, and *Nitrosocosmicus* species were used as outgroups for the Nitrospira *nxrB* and *Nitrospira* AmoA tree, respectively. The phylogenetic calculations included model prediction by ModelFinder (Kalyaanamoorthy et al., 2017), which identified the best‐fit models to be GTR + F + I + G4 and LG + F + G4 for the *Nitrospira nxrB* and *Nitrospira* AmoA trees, respectively. The *Nitrospira* AmoA sequences obtained from sediment of lake HS in an earlier study (Pjevac et al., 2017) are marked with an asterisk (B). Sequences of *nxrB* (A) shown in red or blue are affiliated with comammox *Nitrospira* clade A or B (as identified by Amo gene‐containing, high‐quality MAGs or genomes from cultured strains), respectively. Black and grey stars indicate isolates and enrichment cultures, respectively. Circles at nodes indicate statistical support of branches (1000 bootstrap iterations). The scale bar indicates 30% (A) and 40% (B) estimated sequence divergence.

These two amplicon datasets revealed stable presence of diverse canonical and comammox *Nitrospira* throughout the monitored seasons. High abundance was detected for a *Nitrospira* lineage IV phylotype (OTU‐N1) in both lake sediments and all seasons (Figure 2A, Figure S3). Lineage IV contains *Nitrospira* members from marine and other saline environments including saline‐alkaline lakes (Daebeler et al., 2020; Daims et al., 2016). Whereas OTU‐N1 dominated in lake HS over most of the monitoring period, other OTUs from *Nitrospira* lineages IV and II were also high in abundance throughout the monitoring period in lake US sediment (Figure S3). *Nitrospira* lineage II encompasses canonical NOB and all known comammox *Nitrospira* (Daims et al., 2016).

Interestingly, we detected diverse *Nitrospira amoA* genes from comammox clade A in samples from the entire monitoring period in both lakes (Figure 2B, Figure S4). In contrast, comammox clade B *amoA* was detected only stochastically during four out of the 15 months, and those sequences were not found in replicate sediment samples from either lake. Therefore, we assume that clade B comammox *Nitrospira* were not part of the stable microbial communities in these sediments.

Partially consistent with our hypothesis that seasonal effects (especially fluctuations in pH) would influence the NOB and comammox communities, the *nxrB*‐based *Nitrospira* community structures observed in both lakes differed significantly with pH (Table 1). However, for comammox *Nitrospira amoA* gene‐based communities, we only found a significant correlation between the interacting effects of salinity and pH in lake HS (Table 1). We also found that pH and salinity were strongly autocorrelated with each other and with temperature in both lakes. Thus, we could not deduce the separate influence of these environmental factors on the observed seasonal changes in community structures.

**TABLE 1:** Results of multivariate permutational analyses to correlate differences in *Nitrospira* community composition from monthly samples with pH and salinity^a^

*Nitrospira amoA*, lake HS				
Source of variation	df	SS	*R* ^2^	*p*
Salinity	1	0.05	0.02	0.781
pH	1	0.27	0.08	0.1
Salinity:pH	1	0.47	0.15	**0.023**
*Nitrospira amoA*, lake US				
Source of variation	df	SS	*R* ^2^	*p*
Salinity	1	0.02	0.01	0.769
pH	1	0.06	0.020	0.421
Salinity:pH	1	0.16	0.05	0.108
*Nitrospira nxrB*, lake HS				
Source of variation	df	SS	*R* ^2^	*p*
Salinity	1	0.01	0.01	0.838
pH	1	0.27	0.11	**0.001**
Salinity:pH	1	0.07	0.03	0.193
*Nitrospira nxrB*, lake US				
Source of variation	df	SS	*R* ^2^	*p*
Salinity	1	0.08	0.03	0.070
pH	1	0.13	0.05	**0.008**
Salinity:pH	1	0.08	0.03	0.082

*Note*: Bold values depict statistically significant correlations (*p* < 0.05).

^a^ Note that the factors pH and salinity were autocorrelated and further both correlated with temperature.

### Nitrification rates in pH‐controlled incubations

To study the effects of pH on the nitrite‐oxidizing and comammox communities in more detail, pH‐controlled incubations were performed with sediment slurries and filter‐sterilized water from lakes HS and US. Four different pH levels were maintained in these experiments: neutral (pH 7.6), alkaline (pH 9.0), highly alkaline (pH 10.0), and extremely alkaline (pH 11.0). Supplementary Table S3 lists the measured environmental parameters in the water and sediment samples prior to the start of the incubations.

**FIGURE 3:**
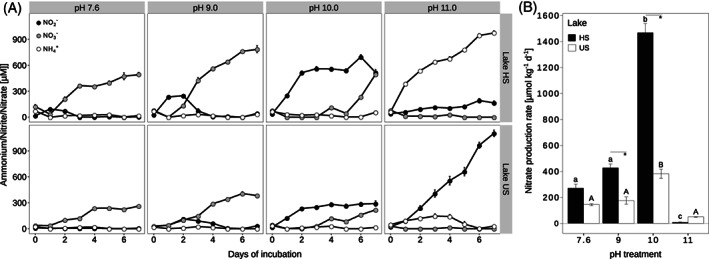
Nitrification activity in pH‐controlled microcosm incubations. (A) Concentrations of nitrite, nitrate, and ammonium in the microcosms during the incubations. The data are split according to the pH treatments. Data points represent means of replicate microcosms (*n* = 4). (B) Gross nitrifications rates determined during the last 24 h of the microcosm incubations. The bars represent means of replicate microcosms (*n* = 4) with standard errors. Lower‐ and upper‐case letters indicate significant differences between microcosms for the same lake HS and US, respectively, while asterisks show significant differences between microcosms for different lakes at a given pH condition. HS, lake Herrensee; US, lake Unterer Stinkersee. Error bars (s.e.m. in both panels) are not visible if smaller than symbols.

Net nitrate accumulation was highest at pH 9 for both lakes, with 737 ± 40 μM and 384 ± 11 μM measured after 7 days of incubation in sediment from lake HS and US, respectively (Figure 3A). While net nitrate production was detected within 2 days at pH 7.6 and pH 9, it was not observed during the first 3 days at pH 10 and not detected at all at pH 11 (Figure 3A). Conversely, net nitrite production was strongest at pH 10 and 11, with highest concentrations reached in lake US incubations at pH 11 (up to 1103 ± 37 μM on Day 7). Ammonium accumulation was detected at pH 11 only. Ammonium accumulated to high concentrations in lake HS incubations (946 ± 20 μM) but transiently reached only considerably lower concentrations in lake US incubations (max. 140 μM) (Figure 3A).

To assess the gross nitrification rates after the microbial communities had adapted to the pH treatments, ^15^ N‐labelled nitrate was added to all microcosms on day six. The gross nitrate production rates were highest at pH 10, with lake HS sediment exhibiting significantly higher rates (1468 *±* 70 μmol nitrate kg^−1^ d^−1^) than lake US sediment (382 *±* 36 μmol nitrate kg^−1^ d^−1^) (Figure 3B). The lowest gross nitrate production rates were determined for the pH 11 treatments. Particularly in lake HS incubations, nitrate production was barely detectable at this extremely alkaline pH (lake HS: 8 *±* 3 μmol nitrate kg^−1^ d^−1^ and lake US: 50 ± 1 μmol nitrate kg^−1^ d^−1^; Figure 3B).

### Community structures of present and active *Nitrospira* in pH‐controlled incubations

At the end of the pH‐controlled incubation experiment, the present and active ammonia and nitrite oxidizers in the microcosms were identified by amplicon sequencing of the detectable *amoA* and *nxrB* genes and transcripts. We detected only *amoA* genes of comammox *Nitrospira* and of AOA, although we also screened for beta‐ and gammaproteobacterial AOB (Table S3). With only five *Nitrososphaerales* OTUs detected, the AOA community was low in diversity in both lake sediments (Figure S5). More details on the *Nitrososphaereales* community are provided in the Supplementary Note S1 and in Figures S5, S6, and S11.

Overall, genes and transcripts of the comammox *Nitrospira amoA* OTUs CMX1, CMX2, CMX3, and CMX2 and CMX10 showed the highest abundances at all tested pH levels in the lake HS and US incubations, respectively (Figure S7). After 7 days of incubation, the *amoA* gene‐based comammox *Nitrospira* communities of most pH treatments differed from the starting community (Day 0) in the lake US incubations. For lake HS, only the pH 10 and 7.6 communities differed significantly from the starting community composition (Figure S8A,C; principal coordinates analysis, *p* ≤ 0.05). The part of the comammox *Nitrospira* community that actively transcribed *amoA* changed significantly after 7 days at all pH levels and for both lakes. The only exception was the pH 11 incubations of lake US, where no significant change of the *amoA*‐transcribing comammox community was observed (Figure S8B,D; principal coordinates analysis, *p* ≤ 0.05). Furthermore, many active comammox *Nitrospira* communities significantly differed from each other after 7 days of incubation between the pH treatments (Figure S8B,D; principal coordinates analysis, *p* ≤ 0.05).

As expected from the results of the seasonal sampling, the only detectable nitrite oxidizers in the pH‐controlled incubations were affiliated with the genus *Nitrospira*. Lake HS sediment showed a strong dominance of *Nitrospira nxrB* OTU‐N1 in both gene and transcript abundance at all pH treatments except at pH 11 (Figure S9A). In the lake US incubations, *Nitrospira nxrB* OTUs N1, N2, N3, and N6 showed the highest *nxrB* gene abundances at all pH levels. However, while *Nitrospira nxrB* OTUs N1 and N6 clearly dominated the transcript‐based communities at pH 11 and 10, respectively, at pH 9 and 7.6, the transcripts of both OTUs N1 and N3 showed highest abundances (Figure S9B). The *Nitrospira nxrB* gene‐based communities did not change significantly in the lake US incubations, while the profiles of pH treatments 7.6, 9, and 10 from the lake HS incubations transitioned significantly from the community profiles present at start of the experiment (Figure S10A,C). Similar to the observation made for the *amoA* of comammox *Nitrospira*, the *nxrB* transcript‐based *Nitrospira* community profiles demonstrated a stronger divergence than the gene‐based communities, especially in lake US where the *nxrB* transcript‐based communities from all pH treatments were significantly different from each other (Figure S10B,D).

## DISCUSSION

### Seasonal dynamics of canonical and comammox *Nitrospira*

Previous studies cultured nitrifiers from other extreme, alkaline and saline systems to analyse their growth characteristics or applied cultivation‐independent approaches to explore the distribution and activity of nitrifiers in situ in such habitats. The detected organisms were closely related to alkalitolerant *Nitrosomonas*, *Nitrosopumilaceae*, *Nitrobacter*, and *Nitrospira* (Carini & Joye, 2008; Daebeler et al., 2020; Lanzén et al., 2013; Sorokin & Kuenen, 2005). However, no representatives of the first three groups were detected in the saline‐alkaline lakes in this study. Instead, we identified members of the *Nitrososphaerales*, comammox *Nitrospira*, and canonical nitrite‐oxidizing *Nitrospira* as the dominant nitrifiers.

The *Nitrospira nxrB* phylotype with the highest abundance throughout the 15‐months observation period in both lakes, OTU‐N1, was closely affiliated with ‘*Ca*. Nitrospira alkalitolerans’ (Figure 2A). This nitrite oxidizer from *Nitrospira* lineage IV had been enriched from another saline‐alkaline lake located close to the two lakes studied here, and its genomic analysis had revealed multiple adaptations to saline and alkaline conditions (Daebeler et al., 2020). In general, *Nitrospira* lineage IV comprises NOB adapted to elevated salinity and alkalinity (Bayer et al., 2021; Daebeler et al., 2020; Daims et al., 2016; Haaijer et al., 2013; Off et al., 2010; Watson et al., 1986). Since lineage IV does not contain any known comammox organism, and because ‘*Ca*. Nitrospira alkalitolerans’ is also a canonical nitrite oxidizer (Daebeler et al., 2020), we assume that OTU‐N1 represents abundant canonical NOB well‐adapted to the environmental conditions in the studied lakes.

Interestingly, we also detected several clade A comammox *Nitrospira amoA* OTUs in the sediment of both lakes at all studied months. Hence, clade A comammox *Nitrospira* are stable members of the indigenous nitrifier community likely contributing to nitrification in these saline‐alkaline systems. Especially comammox *Nitrospira* OTUs CMX1, CMX2 and CMX10 displayed high abundances (Figure S4). These phylotypes did not affiliate closely with *amoA* sequences from physiologically characterized comammox *Nitrospira* strains. Considering the hypertrophic, saline‐alkaline conditions in the two lakes and that these OTUs clustered with sequences obtained in an earlier study (Pjevac et al., 2017) from lake HS as well as from tidal flat sediment and activated sludge (Figure 2B), we assume that they represent comammox strains tolerant to elevated salinity, alkalinity, and hypertrophic conditions. Comammox *Nitrospira* clade B phylotypes were scarce, similar as observed in saline estuary sediments (Zhao et al., 2021).

At the beginning of this study, we had anticipated seasonal effects on the sediment nitrite oxidizer community structure that would largely be evident as effects of the fluctuating pH (see Introduction). Unexpectedly, a relationship with pH was only observed for the *nxrB* gene‐based *Nitrospira* communities. This result contrasts with observations made in other moderately saline environments such as salt marshes and coastal wetlands (Sun et al., 2020; Wang et al., 2021), where pH was identified as one of the strongest environmental factors shaping comammox clade A communities. The absence of any correlation between the *amoA*‐based community structures and pH may indicate a particularly broad tolerance range for pH of the comammox *Nitrospira* populations in the lakes studied here, which display strong seasonal pH fluctuations (Figure 1A). However, since comammox *Nitrospira* also possess *nxrB*, the *nxrB* amplicon dataset will most likely have encompassed comammox and canonical nitrite‐oxidizing *Nitrospira* populations. Therefore, we conclude that the results obtained for *nxrB* and *amoA* gene‐based *Nitrospira* communities indicate that in contrast to some canonical *Nitrospir*a, some comammox *Nitrospira* may be less affected by pH fluctuations in these habitats. Yet, since pH and salinity were correlated with each other as well as with temperature, we cannot confidently dissect the contributions of these factors to seasonal shifts in the canonical and comammox *Nitrospira* community structures. Nevertheless, the results of the microcosm incubation experiments at different pH, but with constant salinity and temperature, demonstrated that comammox and canonical *Nitrospira* were influenced by pH in both community structure and activity (see below and Results).

### Effects of pH on nitrification and canonical and comammox *Nitrospira*

For both lakes, the highest gross nitrification rates were measured in sediment incubated at pH 10 (Figure 3B). This result contrasts our hypothesis that the highest nitrification rates would occur at pH levels close to the average in situ pH (pH 9) of the lakes (see Introduction). Active nitrification has been observed in incubations of soda lake sediments (Sorokin, 1998), and nitrification rates have been shown to positively correlate with pH in soils (Gilmour, 1984; Kyveryga et al., 2004; White & Reddy, 2003) and in a saline‐alkaline wetland (Bai et al., 2010). However, in those studies, the pH levels did not exceed pH 8, the rates were substantially lower, and nitrification had been stimulated by ammonium addition. Possibly, the fast gross nitrate production rates we observed at pH 10 were not only caused by the presence of nitrifiers able to withstand this elevated pH but also fuelled by the higher availability of ammonia versus ammonium above pH 9.5 at the incubation temperature of 28°C. Moreover, we did not add ammonium but measured the nitrification rates based on mineralization within the microcosms as the only source of ammonia. Thus, the obtained rates should reflect the in situ situation quite well. However, if high pH regimes inhibited mineralization more than the nitrifiers, the rates would still underestimate the potential activity of the nitrifier community. In general, the gross nitrate production rates of both lakes observed here were much higher than expected and are, to the best of our knowledge, the highest rates determined so far for a natural aquatic system. At pH 10, the lake HS and US sediment gross nitrate production rates were substantially higher than rates determined in other aquatic habitats, which also were hypertrophic and/or saline environments (Carini & Joye, 2008; Chen et al., 2023; Dai et al., 2008; Hall, 1986; Hampel et al., 2018; Sanders & Laanbroek, 2018). Even though the comparison with gross nitrate production rates determined in other studies is not straightforward due to differences in methodology and normalization, our results testify to the presence of well‐adapted nitrifiers with a unique ecophysiology that are involved in extraordinarily high conversion rates of ammonia/ammonium to nitrate in these extreme systems.

The high nitrate production rates determined here are especially interesting, as the only detectable known ammonia oxidizers present in these not only highly alkaline but also hypertrophic environments were comammox *Nitrospira* and AOA from the *Nitrosophaerales*. Kinetic properties vary considerably among different AOA (Jung et al., 2022) and especially non‐marine members of the *Nitrososphaerales* display relatively low ammonia affinities but higher ammonia oxidation rates than many other AOA (Jung et al., 2022; Kits et al., 2017). Hence, the occurrence and activity of *Nitrososphaerales* in the saline‐alkaline lakes (Figure S5 and S6) appear to be consistent with hypertrophic conditions. The only two kinetically characterized comammox *Nitrospira* species have a very high affinity for ammonia and low ammonia oxidation rates (Jung et al., 2022; Kits et al., 2017; Sakoula et al., 2021). Thus, the high diversity and activity of comammox *Nitrospira* in the studied saline‐alkaline lake sediments with their high ammonium content (>100 μM, Table S2) were unexpected. However, kinetic data from comammox *Nitrospira* are still very limited, and the strains in the studied lake sediments seem to represent comammox organisms that are competitive under non‐oligotrophic conditions. Alternatively, the alkaline and fluctuating pH in the lakes might confer a selective advantage to well‐adapted comammox strains, which enables them to outcompete other ammonia oxidizers despite of kinetic constraints.

At pH 10, net nitrate production was only observed after 4 days of incubation, whereas nitrite accumulated from the beginning of the incubations (Figure 3A). In soils and marine systems, nitrite accumulation is often observed under conditions of transient pH increases, temperature shifts, and low oxygen, and has been explained by different niche adaptations of the ammonia and nitrite oxidizers (Bristow et al., 2015; Burns et al., 1995; Duan et al., 2020; Heiss & Fulweiler, 2016; Maharjan & Venterea, 2013; Schaefer & Hollibaugh, 2017; Taylor et al., 2019). Transient nitrite accumulation has also been observed at neutral pH values for the only available pure comammox culture, *Nitrospira inopinata*, and in comammox *Nitrospira* dominated systems. This phenomenon has been attributed to differences in enzyme kinetics and oxygen tolerance between the ammonia and nitrite oxidation steps in comammox (Kits et al., 2017; Wang et al., 2020). The strong uncoupling of nitrification observed at high pH in our study suggests that the ammonia oxidisers coped better with the elevated alkalinity than the nitrite oxidisers. It remains unknown whether ammonia oxidation at pH 11 was performed by AOA and/or by comammox *Nitrospira* with a reduced nitrite oxidation activity. Uncoupling of the two nitrification steps in comammox *Nitrospira* might theoretically be explained by pH‐mediated changes of the nitrite oxidation kinetics, by pH‐triggered downregulation of the genes for nitrite oxidation, or by damage of the nitrite‐oxidizing enzymatic machinery due to a high pH. These possibilities remain hypothetical, because the data obtained in this study did not allow us to conclusively determine the relative contributions of the different ammonia oxidisers to the observed ammonia oxidation activity. Additionally, one could speculate that a lag phase was needed to express pH stress response mechanisms that were vital especially for nitrite oxidation by canonical *Nitrospira*. Such a delay could also have caused the observed uncoupling of nitrification at pH 11. Previously, we identified an impressive suite of genomic adaptations to alkalinity in the canonical NOB ‘*Ca*. Nitrospira alkalitolerans’ (Daebeler et al., 2020), which had been enriched from the saline‐alkaline sediment of a lake in close vicinity to the lakes of this study. Furthermore, the strong tendency of some *Nitrospira* strains to form microcolonies embedded in extracellular polymeric matrix (Daebeler et al., 2020; Spieck et al., 2006) could contribute to protection from harsh environmental conditions. It appears plausible that these or similar adaptations are shared by other canonical and comammox *Nitrospira* in the lakes, but it remains unknown whether they are more important for nitrite than for ammonia oxidation.

We also hypothesized that at different pH levels, different nitrifiers would be active and responsible for nitrification, thereby indicating niche differentiation (see Introduction). Confirming this hypothesis, we found that many community profiles of transcriptionally active comammox and canonical *Nitrospira* were significantly different between pH levels, in nearly all cases more so than the profiles of the total communities as based on gene abundances (Figure S8 and S10).

Additionally, the collected data allowed us speculate about pH preferences of specific *Nitrospira* phylotypes. While the dominant active comammox *Nitrospira* phylotypes were identical across all pH treatments, for the canonical *Nitrospira* a stronger influence of pH was apparent. For example, *nxrB* transcription by the dominant *Nitrospira* phylotype OTU‐N1 decreased pronouncedly at pH 11 in the incubated samples from both lakes (Figure S8A), in concordance with a significantly decreased gross nitrate production (Figure 3B). This indicates that the nitrite‐oxidizing activity of this phylotype may be compromised above pH 10. In a previous study, a limit of activity above pH 10 was also observed for nitrite‐oxidizing *Nitrospira* enrichments from lake sediments of the same area (Daebeler et al., 2020). Two phylotypes were found to increase in abundance at pH 11: *Nitrospira* OTU‐N6 and comammox *Nitrospira* OTU‐CMX2 showed increased expression of *nxrB* and *amoA*, respectively, in lake US incubations at pH 11 (Figures S8B and S9B). These phylotypes may represent *Nitrospira* strains well adapted to extremely alkaline conditions, albeit with rather low nitrification rates at pH 11 (Figure 3B).

While pH did not seem to have an effect on the seasonally monitored comammox *Nitrospira* community structures, the comammox populations in the microcosm incubations were influenced by pH. A plausible cause of this discrepancy could be that during the seasonal monitoring, pH was measured in the water overlaying the lake sediment and comammox organisms in the sediments might have experienced different pH conditions. In contrast, in the microcosm experiment, the pH was adjusted and measured directly in the sediment‐water slurry. Thus, the results of the microcosm incubations do not fully explain the observed environmental dynamics. Adaptations of comammox *Nitrospira* to high alkalinity are virtually unexplored, but the results of this study suggest that comammox can play a pivotal role for nitrification in alkaline ecosystems. Generally, a broad pH tolerance of some comammox *Nitrospira* is likely a selective advantage in any habitat displaying pH fluctuations between neutral and alkaline conditions.

## AUTHOR CONTRIBUTIONS

**Anne Daebeler:** Conceptualization (equal); data curation (lead); formal analysis (lead); funding acquisition (equal); investigation (lead); methodology (lead); visualization (lead); writing – original draft (lead); writing – review and editing (lead). **Queralt Guell‐Bujons:** Funding acquisition (supporting); methodology (supporting); writing – review and editing (supporting). **Maria Mooshammer:** Conceptualization (supporting); data curation (equal); formal analysis (equal); methodology (equal); writing – review and editing (supporting). **Thomas Zechmeister:** Data curation (supporting); formal analysis (supporting); methodology (supporting); writing – review and editing (supporting). **Craig W. Herbold:** Data curation (supporting); formal analysis (supporting); methodology (supporting); writing – review and editing (supporting). **Andreas Richter:** Resources (supporting); supervision (supporting); writing – review and editing (supporting). **Michael Wagner:** Resources (supporting); supervision (supporting). **Holger Daims:** Conceptualization (equal); funding acquisition (equal); resources (equal); supervision (supporting); writing – review and editing (equal).

## CONFLICT OF INTEREST

The authors declare that they have no conflict of interest.

## Supporting information


**Figure S1.** Map of the sampled saline‐alkaline lakes showing the location of the sampling region in Austria (a) and the geographic location of the sampled lakes in the national park ‘Neusiedler See‐Seewinkel’, Burgenland, Austria (b). The lakes sampled in this study are shown in blue with the corresponding identifier abbreviation next to them. This figure is modified from Daebeler et al. (2020).
**Figure S2.** Measured pH values in the pH‐controlled microcosm incubations. The top panels show the pH in sediment slurries from lake Herrensee (HS), and the lower panels show the pH values for slurries from lake Unterer Stinkersee (US). The data are split according to pH treatment. Data points represent means (*n* = 4) with standard errors, which are not visible if smaller than symbol size.
**Figure S3.** Normalized abundances of *Nitrospira nxrB* gene phylotypes detected in triplicate sediment samples from lake HS (A) and lake US (B) over the course of 15 months. *Nitrospira* communities are grouped by time on the *y*‐axis, and OTUs are grouped by phylogenetic affiliation on the *x*‐axis. Grey colour indicates that an OTU was not detected. Lake HS, lake Herrensee; Lake US, lake Unterer Stinkersee; Lin. II, *Nitrospira* lineage II; Lin. IV, *Nitrospira* lineage IV; Uncl., unclassified affiliation within the genus *Nitrospira*; log(Freq), log scale normalized frequency counts.
**Figure S4.** Normalized abundances of *Nitrospira amoA* gene phylotypes detected in triplicate sediment samples from lake Herrensee (A) and lake Unterer Stinkersee (B) over the course of 15 months. *Nitrospira* communities are grouped by time on the y‐axis. Grey colour indicates that an OTU was not detected. Missing replicates resulted in less than three samples per month in some cases and are due to unsuccessful PCR amplification. Lake HS, lake Herrensee; Lake US, lake Unterer Stinkersee; (log) Freq, log scale normalized frequency counts.
**Figure S5.** Phylogenetic maximum likelihood analysis showing the affiliation of *Nitrososphaerales* AmoA protein sequences (obtained by in silico translation of amoA gene sequences), which were retrieved from pH‐controlled incubations of sediments from the saline‐alkaline lakes Herrensee (HS) and Unterer Stinkersee (US), to selected reference sequences. The AmoA sequences of cultured *Nitrosomonas* and the PmoA gene sequences of cultured *Methylocaldum* species were used as outgroups The phylogenetic calculation included model prediction by ModelFinder (Kalyaanamoorthy et al., 2017), which identified the best‐fit model to be mtZOA + G4. Sequences obtained in this study are printed in bold. Black and grey stars indicate isolates and enrichment cultures, respectively. Circles at nodes indicate statistical support of branches (1000 bootstrap iterations). The scale bar indicates 20% estimated sequence divergence.
**Figure S6.** Normalized abundances of *Nitrososphaerales amoA* gene and transcript phylotypes detected in quadruplicate samples from the beginning and after 7 days of pH‐controlled incubations of sediment from lakes HS (a) and US (b). *Nitrososphaerales* communities are grouped by pH treatment on the *y*‐axis. Grey colour indicates that an OTU was not detected. Missing replicates resulted in less than four samples per treatment in some cases and were due to unsuccessful PCR amplification. Lake HS, lake Herrensee; Lake US, lake Unterer Stinkersee; Start, community profiles detected in mixed sediment before the incubation; pH 7.6, pH 9, pH 10 and pH 11, community profiles detected at the end of the incubation in the different pH treatments; log(Freq), log scale normalized frequency counts.
**Figure S7.** Normalized abundances of *Nitrospira amoA* gene and transcript phylotypes detected in quadruplicate samples from the beginning and after 7 days of pH‐controlled incubations of sediment from lakes HS (a) and US (b). *Nitrospira* communities are grouped by pH treatment on the *y*‐axis. Grey colour indicates that an OTU was not detected. Missing replicates resulted in less than four samples per treatment in some cases and were due to unsuccessful PCR amplification. Lake HS, lake Herrensee; Lake US, lake Unterer Stinkersee; Start, community profiles detected in mixed sediment before the incubation; pH 7.6, pH 9, pH 10 and pH 11, community profiles detected at the end of the incubation in the different pH treatments; log(Freq), log scale normalized frequency counts.
**Figure S8.** Principal coordinate analysis (PCoA) constrained to pH treatment depicting the *amoA*‐harbouring *Nitrospira* communities at the beginning and end of the 7 day incubation at different pH levels. Colours depict the pH of the incubation and each point indicates an independently sequenced sample, hence replicates are shown as points of equal colour. Ellipses show 95% confidence intervals, therefore non‐overlapping ellipses indicate significant (*p* ≤ 0.05) differences between respective communities. The pH values of 8.4 and 8.5 (lake Herrensee and lake Unterer Stinkersee, respectively) were determined in freshly sampled sediment and indicate the community composition before the beginning of the incubation, while all other points indicate community composition in samples from the end of the incubation. Communities are based on Bray–Curtis distances computed using *Nitrospira amoA* gene (panels A and C) and transcript (panels B and D) OTUs from sediment of lake Herrensee (panels A and B) and Unterer Stinkersee (panels C and D).
**Figure S9.** Normalized abundances of *Nitrospira nxrB* gene phylotypes detected in quadruplicate samples from the beginning and after 7 days of pH‐controlled incubations of sediment from lakes HS (a) and US (b). *Nitrospira* communities are grouped by pH treatment on the *y*‐axis and OTUs are grouped by phylogenetic affiliation on the *x*‐axis. Grey colour indicates that an OTU was not detected. Missing replicates resulted in less than four samples per treatment in some cases and are due to unsuccessful PCR amplification. Lake HS, lake Herrensee; Lake US, lake Unterer Stinkersee; Start, community profiles detected in mixed sediment before the incubation; pH 7.6, pH 9, pH 10 and pH 11, community profiles detected at the end of the incubation in the different pH treatments; log(Freq), log scale normalized frequency counts.
**Figure S10.** Principal coordinate analysis (PCoA) constrained to pH treatment depicting the *nxrB*‐harbouring *Nitrospira* communities at the beginning and end of the 7 day incubation at different pH levels. Colours depict the pH of the incubation and each point indicates an independently sequenced sample, hence replicates are shown as points of equal colour. Ellipses show 95% confidence intervals, therefore non‐overlapping ellipses indicate significant (*p* ≤ 0.05) differences between respective communities. The pH values of 8.4 and 8.5 (lake Herrensee and lake Unterer Stinkersee, respectively) were determined in freshly sampled sediment and indicate the community composition before the beginning of the incubation, while all other points indicate community composition in samples from the end of the incubation. Communities are based on Bray–Curtis distances computed using *Nitrospira nxrB* gene (panels A and C) and transcript (panels B and D) OTUs from sediment of lake Herrensee (panels A and B) and Unterer Stinkersee (panels C and D).
**Figure S11.** Principal coordinate analysis (PCoA) constrained to pH treatment depicting the *amoA*‐harbouring *Nitrososphareales* communities at the beginning and end of the 7 day incubation at different pH levels. Colours depict the pH of the incubation and each point indicates an independently sequenced sample, hence replicates are shown as points of equal colour. Ellipses show 95% confidence intervals, therefore non‐overlapping ellipses indicate significant (*p* ≤ 0.05) differences between respective communities. The pH values of 8.4 and 8.5 (lake Herrensee and lake Unterer Stinkersee, respectively) were determined in freshly sampled sediment and indicate the community composition before the beginning of the incubation, while all other points indicate community composition in samples from the end of the incubation. Communities are based on Bray–Curtis distances computed using *Nitrososphareales amoA* gene (panels A and C) and transcript (panels B and D) OTUs from sediment of lake Herrensee (panels A and B) and Unterer Stinkersee (panels C and D).

**Data S1.** Supporting Information.

**Table S1.** Measured values of nitrate concentration and 15 N/14 N in nitrate in microcosm incubations 2.5, 24 and 48 h after the addition of 15 N‐nitrate on Day 6

**Table S2.** Primers and PCR profiles used in this study.

**Table S3.** Environmental conditions in sediment and water samples used for incubation experiment

## Data Availability

The raw, demultiplexed fastq files were deposited at the European Nucleotide Archive (ENA) database under study accession numbers PRJEB47424, PRJEB47446, PRJEB47390, PRJEB47406, and PRJEB47407 for the data sets of *amoA* of comammox *Nitrospira* from seasonal samples, *nxrB* of canonical and comammox *Nitrospira* from seasonal samples, *amoA* of comammox *Nitrospira* from the pH‐controlled incubations, *nxrB* of canonical and comammox *Nitrospira* from the pH‐controlled incubations, and *amoA* of *Nitrososphaerales* from the pH‐controlled incubations, respectively.
