# Supplementary material for: Rapid nitrification involving comammox and canonical Nitrospira at extreme pH in saline‐alkaline lakes
Source: Environ Microbiol. 2023 Feb 3;25(5):1055–67. doi: 10.1111/1462-2920.16337 (PMC10947350; doi:10.1111/1462-2920.16337)
Supplement: Supplementary file 1 — Figure S1. Map of the sampled saline‐alkaline lakes showing the location of the sampling region in Austria (a) and the geographic location of the sampled lakes in the national park ‘Neusiedler See‐Seewinkel’, Burgenland, Austria (b). The lakes sampled in this study are shown in blue with the corresponding identifier abbreviation next to them. This figure is modified from Daebeler et al. (2020). Figure S2. Measured pH values in the pH‐controlled microcosm incubations. The top panels show the pH in sediment slurries from lake Herrensee (HS), and the lower panels show the pH values for slurries from lake Unterer Stinkersee (US). The data are split according to pH treatment. Data points represent means (n = 4) with standard errors, which are not visible if smaller than symbol size. Figure S3. Normalized abundances of Nitrospira nxrB gene phylotypes detected in triplicate sediment samples from lake HS (A) and lake US (B) over the course of 15 months. Nitrospira communities are grouped by time on the y‐axis, and OTUs are grouped by phylogenetic affiliation on the x‐axis. Grey colour indicates that an OTU was not detected. Lake HS, lake Herrensee; Lake US, lake Unterer Stinkersee; Lin. II, Nitrospira lineage II; Lin. IV, Nitrospira lineage IV; Uncl., unclassified affiliation within the genus Nitrospira; log(Freq), log scale normalized frequency counts. Figure S4. Normalized abundances of Nitrospira amoA gene phylotypes detected in triplicate sediment samples from lake Herrensee (A) and lake Unterer Stinkersee (B) over the course of 15 months. Nitrospira communities are grouped by time on the y‐axis. Grey colour indicates that an OTU was not detected. Missing replicates resulted in less than three samples per month in some cases and are due to unsuccessful PCR amplification. Lake HS, lake Herrensee; Lake US, lake Unterer Stinkersee; (log) Freq, log scale normalized frequency counts. Figure S5. Phylogenetic maximum likelihood analysis showing the affiliation of Nitrososphaer [file EMI-25-1055-s005.docx]

**Supplemental figures**

**Figure S1.** Map of the sampled saline-alkaline lakes showing the location of the sampling region in Austria (a) and the geographic location of the sampled lakes in the national park “Neusiedler See-Seewinkel”, Burgenland, Austria (b). The lakes sampled in this study are shown in blue with the corresponding identifier abbreviation next to them. This figure is modified from Daebeler *e**t al.* (2020).

Daebeler, A., Kitzinger, K., Koch, H., Herbold, C.W., Steinfeder, M., Schwarz, J., et al. (2020) Exploring the upper pH limits of nitrite oxidation: diversity, ecophysiology, and adaptive traits of haloalkalitolerant *Nitrospira*. *ISME J* **14**: 2967–2979.

**Figure S2.** Measured pH values in the pH-controlled microcosm incubations. The top panels show the pH in sediment slurries from lake Herrensee (HS), and the lower panels show the pH values for slurries from lake Unterer Stinkersee (US). The data is split according to pH treatment. Data points represent means (*n*=4) with standard errors, which are not visible if smaller than symbol size.

**Figure S3.** Normalized abundances of *Nitrospira nxrB* gene phylotypes detected in triplicate sediment samples from lake HS (A) and lake US (B) over the course of 15 months. *Nitrospira* communities are grouped by time on the *y*-axis, and OTUs are grouped by phylogenetic affiliation on the *x*-axis. Grey color indicates that an OTU was not detected. Lake HS, lake Herrensee; Lake US, lake Unterer Stinkersee; Lin. II, *Nitrospira* lineage II; Lin. IV, *Nitrospira* lineage IV; Uncl., unclassified affiliation within the genus *Nitrospira*; log(Freq), log scale normalized frequency counts.

**Figure S4.** Normalized abundances of *Nitrospira amoA* gene phylotypes detected in triplicate sediment samples from lake Herrensee (A) and lake Unterer Stinkersee (B) over the course of 15 months. *Nitrospira* communities are grouped by time on the y-axis. Grey color indicates that an OTU was not detected. Missing replicates resulted in less than three samples per month in some cases and are due to unsuccessful PCR amplification. Lake HS, lake Herrensee; Lake US, lake Unterer Stinkersee; (log) Freq, log scale normalized frequency counts.

**Figure S5.** Phylogenetic maximum likelihood analysis showing the affiliation of *Nitrososphaerales* *AmoA* protein sequences (obtained by *in silico* translation of *amoA* gene sequences), which were retrieved from pH-controlled incubations of sediments from the saline-alkaline lakes Herrensee (HS) and Unterer Stinkersee (US), to selected reference sequences. The AmoA gene sequences of cultured *Nitrosomonas* and the PmoA gene sequences of cultured *Methylocaldum* species were used as outgroups The phylogenetic calculation included model prediction by ModelFinder (Kalyaanamoorthy *et al.*, 2017), which identified the best-fit model to be mtZOA+G4. Sequences obtained in this study are printed in bold. Black and grey stars indicate isolates and enrichment cultures, respectively. Circles at nodes indicate statistical support of branches (1000 bootstrap iterations). The scale bar indicates 20% estimated sequence divergence.

**Figure S6.** Normalized abundances of *Nitrososphaerales amoA* gene and transcript phylotypes detected in quadruplicate samples from the beginning and after 7 days of pH-controlled incubations of sediment from lakes HS (a) and US (b). *Nitrososphaerales* communities are grouped by pH treatment on the *y*-axis. Grey color indicates that an OTU was not detected. Missing replicates resulted in less than four samples per treatment in some cases and were due to unsuccessful PCR amplification. Lake HS, lake Herrensee; Lake US, lake Unterer Stinkersee; Start, community profiles detected in mixed sediment before the incubation; pH 7.6, pH 9, pH 10 and pH 11, community profiles detected at the end of the incubation in the different pH treatments; log(Freq), log scale normalized frequency counts.

**Figure S7.** Normalized abundances of *Nitrospira amoA* gene and transcript phylotypes detected in quadruplicate samples from the beginning and after 7 days of pH-controlled incubations of sediment from lakes HS (a) and US (b). *Nitrospira* communities are grouped by pH treatment on the *y*-axis. Grey color indicates that an OTU was not detected. Missing replicates resulted in less than four samples per treatment in some cases and were due to unsuccessful PCR amplification. Lake HS, lake Herrensee; Lake US, lake Unterer Stinkersee; Start, community profiles detected in mixed sediment before the incubation; pH 7.6, pH 9, pH 10 and pH 11, community profiles detected at the end of the incubation in the different pH treatments; log(Freq), log scale normalized frequency counts.

**
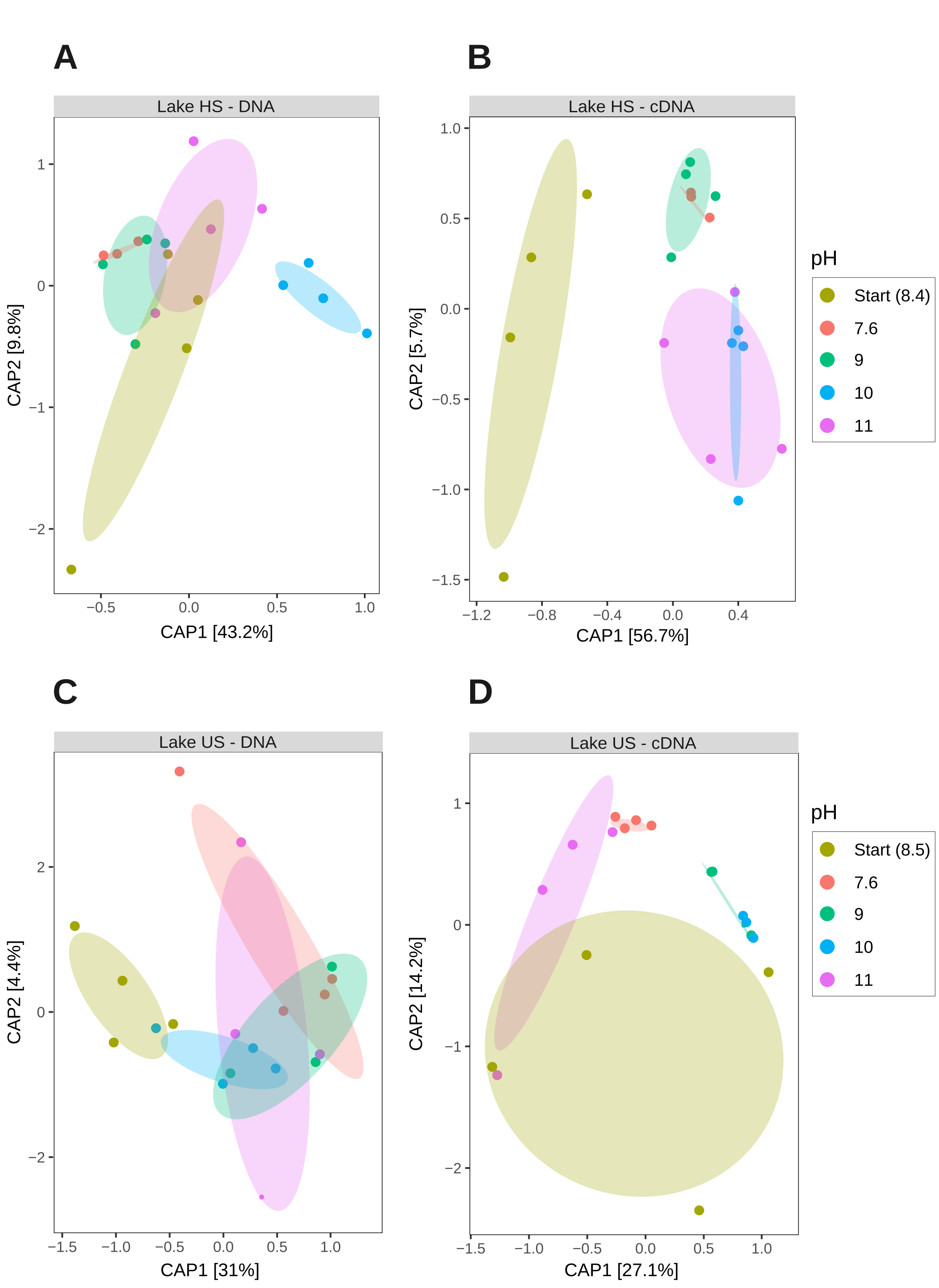
**

**Figure S8** – Principal coordinate analysis (PCoA) constrained to pH treatment depicting the *amoA*-harboring *Nitrospira* communities at the beginning and end of the 7 day incubation at different pH levels. Colors depict the pH of the incubation and each point indicates an independently sequenced sample, hence replicates are shown as points of equal color. Ellipses show 95% confidence intervals, therefore non-overlapping ellipses indicate significant (p≤ 0.05) differences between respective communities. The pH values of 8.4 and 8.5 (lake Herrensee and lake Unterer Stinkersee, respectively) were determined in freshly sampled sediment and indicate the community composition before the beginning of the incubation, while all other points indicate community composition in samples from the end of the incubation. Communities are based on Bray-Curtis distances computed using *Nitrospira* *amoA* gene (panel A and C) and transcript (panel B and D) OTUs from sediment of lake Herrensee (panel A and B) and Unterer Stinkersee (panel C and D).

**Figure S9.** Normalized abundances of *Nitrospira nxrB* gene phylotypes detected in quadruplicate samples from the beginning and after 7 days of pH-controlled incubations of sediment from lakes HS (a) and US (b). *Nitrospira* communities are grouped by pH treatment on the *y*-axis and OTUs are grouped by phylogenetic affiliation on the *x*-axis. Grey color indicates that an OTU was not detected. Missing replicates resulted in less than four samples per treatment in some cases and are due to unsuccessful PCR amplification. Lake HS, lake Herrensee; Lake US, lake Unterer Stinkersee; Start, community profiles detected in mixed sediment before the incubation; pH 7.6, pH 9, pH 10 and pH 11, community profiles detected at the end of the incubation in the different pH treatments; log(Freq), log scale normalized frequency counts.

**
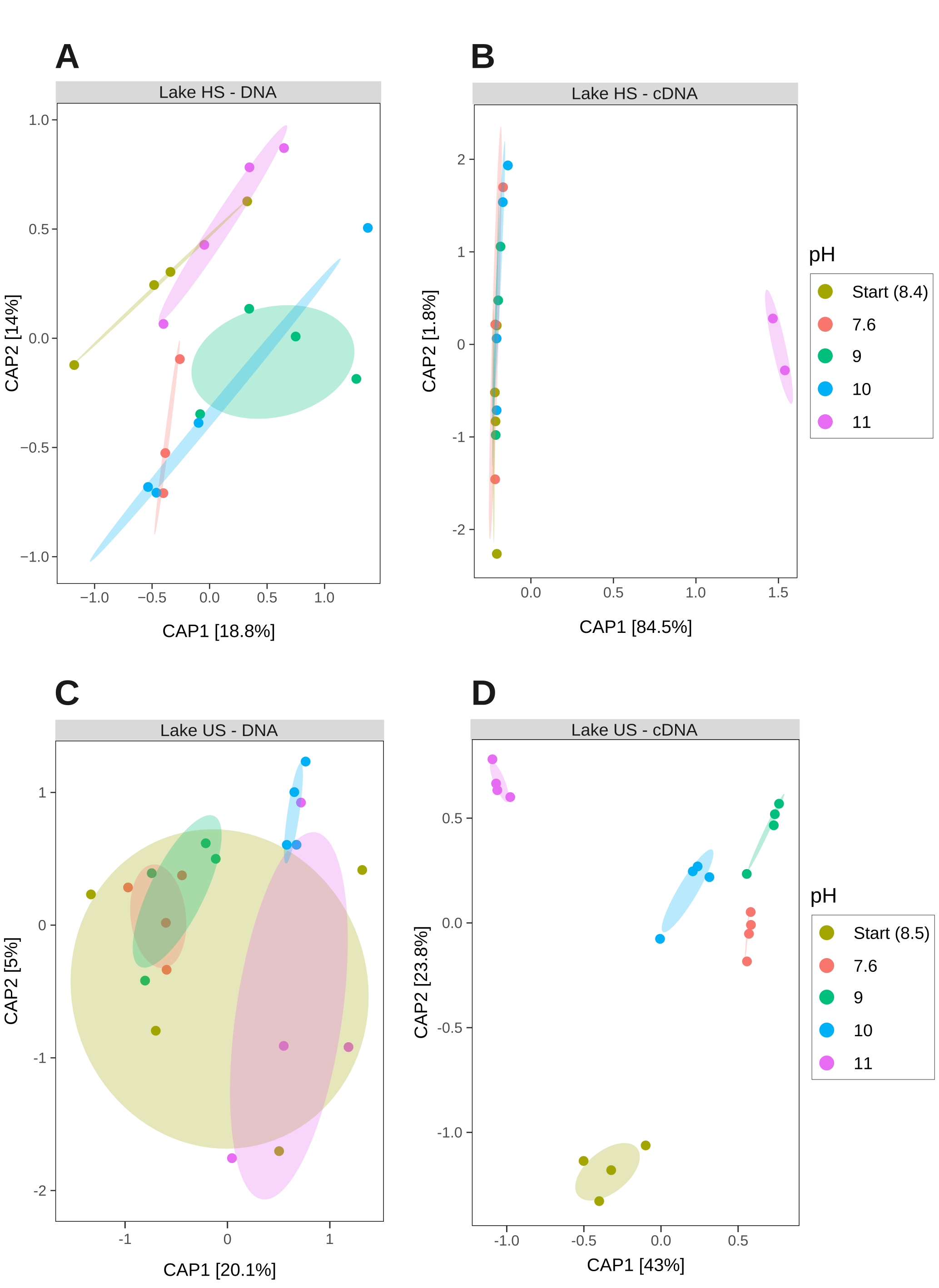
**

**Fig S10 –** Principal coordinate analysis (PCoA) constrained to pH treatment depicting the *nxrB*-harboring *Nitrospira* communities at the beginning and end of the 7 day incubation at different pH levels. Colors depict the pH of the incubation and each point indicates an independently sequenced sample, hence replicates are shown as points of equal color. Ellipses show 95% confidence intervals, therefore non-overlapping ellipses indicate significant (p≤ 0.05) differences between respective communities. The pH values of 8.4 and 8.5 (lake Herrensee and lake Unterer Stinkersee, respectively) were determined in freshly sampled sediment and indicate the community composition before the beginning of the incubation, while all other points indicate community composition in samples from the end of the incubation. Communities are based on Bray-Curtis distances computed using *Nitrospira* *nxrB* gene (panel A and C) and transcript (panel B and D) OTUs from sediment of lake Herrensee (panel A and B) and Unterer Stinkersee (panel C and D).

**
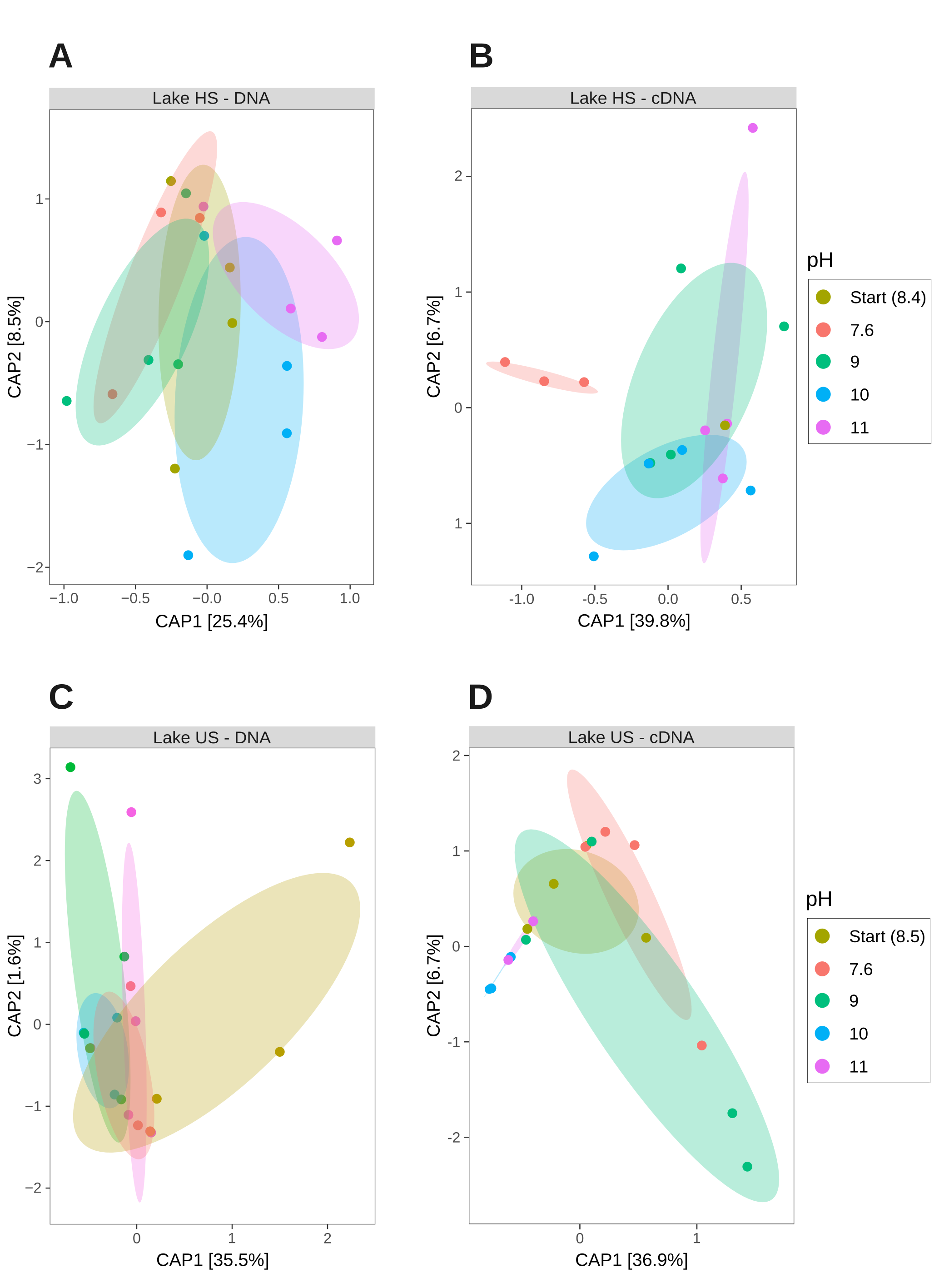
**

**Figure S11** – Principal coordinate analysis (PCoA) constrained to pH treatment depicting the *amoA*-harboring *Nitrososphareales* communities at the beginning and end of the 7 day incubation at different pH levels. Colors depict the pH of the incubation and each point indicates an independently sequenced sample, hence replicates are shown as points of equal color. Ellipses show 95% confidence intervals, therefore non-overlapping ellipses indicate significant (p≤ 0.05) differences between respective communities. The pH values of 8.4 and 8.5 (lake Herrensee and lake Unterer Stinkersee, respectively) were determined in freshly sampled sediment and indicate the community composition before the beginning of the incubation, while all other points indicate community composition in samples from the end of the incubation. Communities are based on Bray-Curtis distances computed using *Nitrososphareales* *amoA* gene (panel A and C) and transcript (panel B and D) OTUs from sediment of lake Herrensee (panel A and B) and Unterer Stinkersee (panel C and D).
