# Supplementary material for: Rapid nitrification involving comammox and canonical Nitrospira at extreme pH in saline‐alkaline lakes
Source: Environ Microbiol. 2023 Feb 3;25(5):1055–67. doi: 10.1111/1462-2920.16337 (PMC10947350; doi:10.1111/1462-2920.16337)
Supplement: Supplementary file 2 — Data S1. Supporting Information. [file EMI-25-1055-s002.docx]

**Supplemental note**

The low number of N*itrososphaerales* phylotypes detected in this study could partially be due to the low recommended phylotype clustering level according to Pester et al. 2011. However, the *Nitrososphaerales* OTUs AOA1 and AOA2 were the dominant AOA phylotypes at all pH levels in both lake sediments, with lake HS also showing high relative abundances of OTU-AOA3 related to the genus *Nitrosocosmicus* (Supp. Fig. S5 and S6). OTU AOA1 most actively transcribed the *amoA* gene at all pH levels, except at neutral pH for lake HS, where transcripts of AOA2 transcripts showed equal relative abundance (Supp. Fig. S6A). The gene- and transcript-based community profiles of *Nitrososphaerales* did not change during the incubations at different pH levels for either lake and remained similar to each other, except for the transcript-based community from pH 7.6 incubations with sediment from lake HS (Supp. Fig. S11).

References:

Pester, M.R., Thomas; Flechl, Stefan;, Gröngröft, A.R., Andreas;, Overmann, J.R.-H., Barbara;, and Loy, A.W., Michael (2011) amoA-based consensus phylogeny of ammonia-oxidizing archaea and deep sequencing of amoA genes from soils of four different geographic regions. *Environ Microbiol* **14**: 525–539.
